# The continuing HIV vaccine saga: is a paradigm shift necessary?

**DOI:** 10.1186/1476-9433-5-2

**Published:** 2006-05-18

**Authors:** Kendall A Smith

**Affiliations:** 1 The Division of Immunology, Department of Medicine, Weill Medical College, Cornell University, New York, NY, 10021, USA

## Abstract

As pointed out in previous editorials, the development of an effective vaccine for the Human Immunodeficiency Virus capable of preventing infection, or even one capable of preventing the Acquired Immunodeficiency Disease Syndrome, has eluded investigators for the past 20 years. Now Reche and Keskin and their co-workers have provided evidence that an entirely new approach, based upon modern bioinformatics methods and skillful *in vitro *immunological experiments, may result in an effective way to prime the T cell immune response of normal individuals against conserved peptide epitopes.

The report by Reche, Keskin and co-workers, entitled "*Elicitation from virus-naïve individuals of cytotoxic T lymphocytes directed against conserved HIV-1 epitopes*", gives one pause, in that in the words of the authors, it suggests that a "paradigm shift" is necessary to develop T cell vaccines capable of protecting uninfected individuals from developing AIDS, should they become infected with HIV-1. This sort of claim has been made before in the two-decade quest to develop an effective HIV vaccine. However, the refreshing aspect of this work is that these investigators show the way to both make and test such a vaccine in humans.

The investigators begin with a first principal of immunology, i.e. the most rational targets of the immune system for vaccine development will be the conserved T cell epitopes of the virus, in that these short amino acid stretches are necessary for the virus to maintain viability and reproductive capacity (i.e. viral "fitness"). To determine which of ~ 199 unique HIV-1 9-mer epitopes shown to elicit CTL responses (thus being processed) could be used, these workers developed bioinformatics tools to determine first which potential epitopes are conserved in all HIV-1 clades. From their analysis, they found that only 37 epitopes (6% of 595 total epitopes catalogued) met their strict criteria.

Next the investigators tackled the difficult problem of HLA I polymorphisms, which further complicates the development of a CTL epitope vaccine that would be broad enough to cover almost all of a diverse population of individuals. They developed an algorithm that computed binding of each of the 37 epitopes to 55 HLA I alleles derived from 5 major ethnicities in the U.S. From these calculations as few as five of the 37 epitopes were predicted to be recognized by ≥ 95% of the population. Moreover, 5-epitope combinations using only 25 of the 37 epitopes were identified that are important either for structural integrity and/or catalytic activity of POL (14), GAG (5), ENV (3), and NEF (3).

To determine whether these epitopes would stimulate a measurable CD8+ T cell response from HIV-positive individuals, peripheral blood mononuclear cells (PBMC) from 47 subjects were activated *in vitro *for 18 hours with the 5 distinct peptide pools followed by assays for INFγ ELISPOTS. In contrast to the expected readily detectable responses from ≥ 95% of the subjects, only 31–45% scored positive, and even then the responses were low, < 1000 Spots/million PBMCs.

Most investigators would have stopped at this stage, assuming that their original hypothesis was not valid, i.e. that these epitopes could not detect immune responses from chronically infected individuals, either because of CTL "exhaustion", or clonal deletion, leaving "holes in the TCR repertoire". However, these investigators decided to switch their studies to HIV-negative individuals, to determine whether it was possible to generate HIV peptide-specific CTL reactive with the selected peptides. First, they created HIV peptide pool-activated long-term CTL lines by priming PBMCs of 10 donors, followed by testing for ELISPOT analysis. Their results were clear-cut and markedly superior to those obtained from the HIV-positive subjects: CTL lines from all donors yielded IFNγ ELISPOTS, most ranging from 1000 to > 6000 SFC/million cells, thereby indicating that normal individuals had lymphocyte precursors capable of recognizing these peptide pools.

Next, they restricted their analysis to HLA A0201, and determined that eight HIV-1 peptides predicted to bind to A0201 could actually do so. As well, they demonstrated that this pool of 8 peptides could activate the proliferation of PBMCs from an A0201 donor, and that these activated cells were capable of secreting IFNγ. Moreover, from 1%–9% of the cells from these long-term CTL lines specifically bound peptide/MHC dimers. Finally, these peptide-generated CTL lines were capable of lysis of both peptide-pulsed target cells, as well as HIV-1 infected cells, thereby indicating their potential immunologic reactivity.

These results do not speak to the reason(s) that cells from HIV-positive individuals were incapable of recognizing and responding to conserved CTL epitopes, so that future experiments exploring the various hypotheses to explain the lack of reactivity are warranted. However, the results obtained with cells from normal, HIV-negative individuals are notable, because they have already identified the conserved epitopes that could be used to construct an HIV vaccine, and they have shown that these epitopes are capable of promoting the expansion of peptide-specific CD8+ T cells from normals to the point that they can lyse HIV-1 infected cells. If this could be achieved *in vivo*, perhaps an effective T cell HIV vaccine could be produced.

